# Individuals Diagnosed With Type 2 Diabetes Mellitus and the Status of Vitamin B12 Deficiency: A Review

**DOI:** 10.7759/cureus.55103

**Published:** 2024-02-27

**Authors:** Pratiksha S Batulwar, Ashish Anjankar

**Affiliations:** 1 Biochemistry, Jawaharlal Nehru Medical College, Datta Meghe Institute of Higher Education and Research, Wardha, IND

**Keywords:** neuropathy, homocystine, metformin, diabetes mellitus, vitamin b12 deficiency

## Abstract

Type 2 diabetes mellitus (T2DM) is a complex metabolic disorder with a multifactorial etiology and a significant global burden. In recent years, emerging evidence has suggested a potential link between T2DM and vitamin B12 deficiency, raising concerns about its impact on disease progression, management, and associated complications. This comprehensive review critically examines the current understanding of the prevalence, risk factors, clinical implications, and management strategies related to vitamin B12 deficiency in individuals diagnosed with T2DM. The review begins by providing an overview of the epidemiology of T2DM and its associated complications, underscoring the need for comprehensive management approaches. Subsequently, it delves into the physiology of vitamin B12, including its sources, absorption mechanisms, and biological functions, laying the groundwork for understanding the potential implications of deficiency in T2DM. A thorough analysis of the literature is conducted to elucidate the prevalence and risk factors of vitamin B12 deficiency in individuals with T2DM, considering factors such as age, duration of diabetes, medication use (e.g., metformin), dietary patterns, and comorbidities. Special attention is given to the role of metformin, the first-line therapy for T2DM, in precipitating or exacerbating vitamin B12 deficiency through mechanisms involving alterations in the gut microbiota and intestinal absorption.

The review further explores the clinical manifestations and diagnostic challenges associated with vitamin B12 deficiency in the context of T2DM, emphasizing the importance of recognizing subtle symptoms and implementing appropriate screening protocols. It discusses the potential implications of vitamin B12 deficiency on glycemic control, diabetic neuropathy, cognitive function, cardiovascular health, and overall quality of life in individuals with T2DM. In addressing the management of vitamin B12 deficiency in T2DM, the review examines various therapeutic strategies, including oral and parenteral supplementation, dietary modifications, and lifestyle interventions. It critically evaluates the evidence supporting routine screening for vitamin B12 deficiency in individuals with T2DM and discusses controversies surrounding optimal supplementation protocols, dosing regimens, and monitoring strategies. Furthermore, the review highlights gaps in current knowledge and identifies areas for future research, such as the long-term effects of vitamin B12 supplementation on clinical outcomes in T2DM, the impact of genetic factors on vitamin B12 metabolism, and the potential role of personalized interventions. Overall, this review consolidates existing evidence and provides insights into the complex relationship between T2DM and vitamin B12 deficiency, aiming to inform clinical practice, enhance patient care, and guide future research endeavors in this important area of metabolic medicine.

## Introduction and background

Vitamin B12, also referred to as cobalamin, is a hydrophilic vitamin crucial for the synthesis of DNA, blood cell formation, and neurological functions. Individuals with insufficient B12 intake might need supplements, and signs of deficiency include headaches and fatigue. Despite B12 being stored in the liver for up to five years, inadequate dietary support can lead to depletion over time [1]. Peripheral neuropathy affects more than half of diabetic individuals, elevating the risk of significant complications such as changes in productivity, infections, fractures, amputations, leg ulcers, and increased healthcare utilization [2]. The physiological impacts of vitamin B12 are manifested through two biochemical processes: the methylation process, converting homocysteine into methionine, and the transformation of succinyl-coenzyme A (CoA) from methyl malonyl-coenzyme A (CoA) [3]. A biochemical process is defined as a sequence of chemical reactions that occur within living organisms, driving essential functions such as metabolism, growth, and reproduction. Methylation is a biochemical process where a methyl group (CH_3_) is added to a molecule, often affecting its function or activity.

Vitamin B12 serves as a co-factor in the methylation process, transforming homocysteine into methionine and thereby participating in the formation of S-adenosylmethionine. A deficiency in vitamin B12 disrupts this metabolic pathway, resulting in higher levels of methylmalonic acid (MMA). Consequently, the production of fatty acids from neuronal membranes is impaired. Vitamin B12 plays a vital function in the production of monoamines, including neurotransmitters such as dopamine and serotonin. The neurocognitive or cognitive symptoms linked to insufficient levels of vitamin B12 arise from a combination of these factors [4]. Neuronal injury resulting from insufficient vitamin B12 manifests as axonal demyelination and, ultimately, cell death. Symptoms include temporary simultaneous spinal cord degeneration, delusions, Alzheimer's disease, and peripheral or autonomous neuropathy. Research suggests a greater frequency of vitamin B12 insufficiency in individuals who have type 2 diabetes (T2D). Research evaluating individuals on metformin suggests that B12 deficiency can occur in 5.8% to 33% of cases. While routine testing for vitamin B12 insufficiency is not universally endorsed by organizations like American Diabetes Association (ADA), National Institute for Health and Care Excellence (NICE), or European Association for the Study of Diabetes (EASD), periodic assessments may be beneficial for those using metformin, particularly if they experience neuropathy or anemia [5]. Long-term use of metformin at doses exceeding 2 g/day for over four years may be linked to a biochemical B12 deficit. However, there is a lack of consensus on threshold values for vitamin B12 insufficiency, with variations in definitions such as those provided by the World Health Organization (WHO). According to the WHO, serum levels above 221 pmol/L are considered "B12 adequacy," levels between 148 and 221 pmol/L indicate "low B12," and levels below 148 pmol/L (200 pg/ml) are deemed "B12 insufficiency" [6].

The key points for this review article are vitamin B12 deficiency, diabetes mellitus (DM), metformin, homocysteine, and neuropathy.

## Review

Diabetes

Diabetes mellitus (DM) is a persistent metabolic condition marked by elevated blood glucose levels due to either an insufficient amount of insulin, insulin resistance caused by dysfunctional cells, or a combination of both factors. There are different identifiable types of diabetes from a clinical perspective, which encompass conditions such as monogenic forms of diabetes (e.g., maturity-onset diabetes of the young or neonatal diabetes), gestational diabetes, and a probable autoimmune variant occurring later in life termed autoimmune diabetes of adulthood with latent onset [7,8]. Traditionally, diabetes has been separated into an autoimmune type that typically develops early in life, type 1 diabetes (T1D), and a non-autoimmune type that usually manifests later, type 2 diabetes (T2D). T2D broadly refers to any diabetes not linked to autoimmunity or single-gene causes. There's growing acknowledgment that T2D may involve various physiological conditions [9].

Type 1 Diabetes Mellitus (T1DM) 

T1D, also known as insulin-dependent, juvenile, or childhood-onset diabetes, this condition is marked by insufficient insulin production, requiring daily insulin therapy. In 2017, around nine million individuals were impacted by T1D, with a predominant concentration in affluent regions. The causes and preventive measures for T1D remain poorly comprehended. Symptoms of T1D encompass frequent urination, thirst, increased appetite, weight loss, changes in vision, and fatigue, which may suddenly manifest [10].

Type 2 Diabetes Mellitus (T2DM)

T2DM is marked by the body's ineffectiveness in utilizing insulin and is alternatively referred to as non-insulin-dependent or adult-onset diabetes. This variety impacts over 95% of people with diabetes and is primarily linked to elevated BMI and insufficient exercise [11]. Despite indicators sharing similarities with those of T1D, they are typically less severe. Consequently, the diagnosis of T2D might be delayed by several years, potentially leading to complications already being present. Initially observed mostly in adults, T2D is increasingly being diagnosed in children too.

Consequences of diabetes mellitus

**Figure 1:**
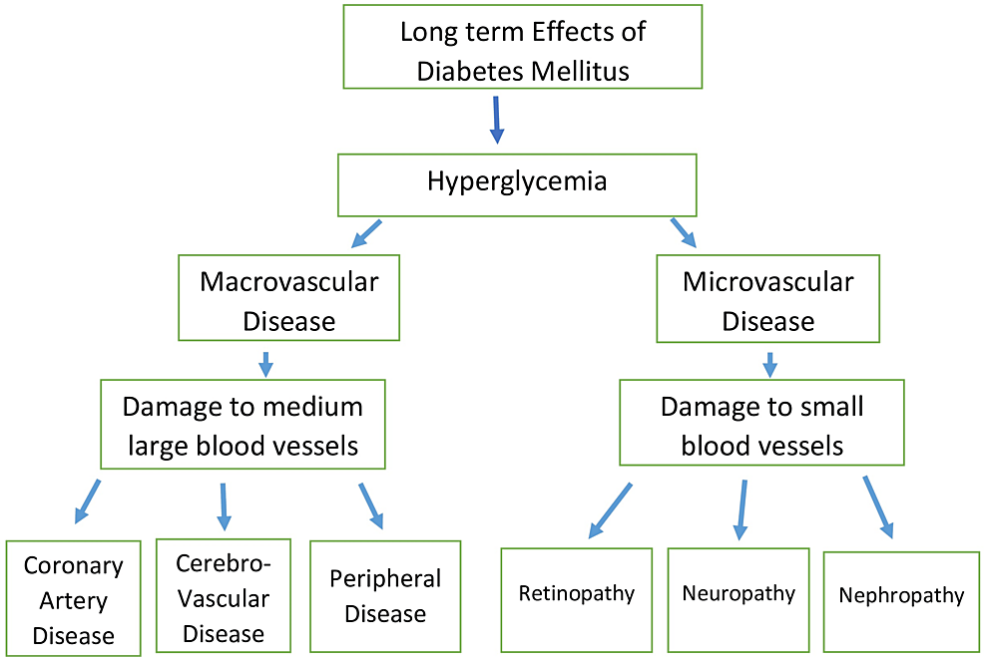
Consequences of diabetes mellitus. Note: This image is the author's own creation.

Both forms of diabetes mellitus (DM) are linked to numerous complications that impact various essential networks within the individual. Diabetes is associated with persistent harm to either the major or minor vascular systems, known as both the large-scale vascular network and the small-scale vascular network [12]. While injuries to the large blood vessel system, like the arteries of the heart and brain, are the main trigger of mortality in individuals with type 2 diabetes (T2D), the more prevalent and impactful harm caused by hyperglycemia affects the microvascular system, leading to issues in the kidneys, eyes, and nerves, significantly impacting mortality rates [13]. The long-term consequences of type 2 diabetes are diagrammatically explained in Figure 1. 

Macrovascular Complications

Cardiovascular disease (CVD) stands as the primary reason for mortality for many subjects with diabetes. Macrovascular problems primarily stem from the narrowing of arteries and veins due to atherosclerosis, leading to conditions like cardiovascular disease and cerebrovascular disease. Diabetes is a substantial and controllable standalone contributing factor for the onset of cardiovascular disease (CVD). Brain-related conditions like stroke and ischemia impact a considerable proportion (20%-40%) of diabetic individuals, primarily resulting from atherosclerosis-induced narrowing in intracranial vessels and the carotid artery. Around 80% of individuals with diabetes aged 65 and older succumb to heart disease, with approximately 16% facing mortality due to stroke [7,11]. Another significant complication is peripheral artery disease (PAD), which is an ailment of the lower extremities characterized by atherosclerotic blockages and posing a notable risk of limb amputation. Diabetes heightens the risk of PAD development on its own, with diabetic individuals often experiencing severe limb ischemia, a severe form of peripheral artery disease (PAD) stage characterized by discomfort at rest and enduring inability [14].

Microvascular Complications

Poorly managed hyperglycemia can result in complications in small blood vessels, such as microangiopathy, which impacts small blood vessels like capillaries, leading to complications such as nephropathy, neuropathy, and retinopathy [15]. Diabetic nephropathy, commonly referred to as diabetic kidney disease, is a common issue characterized by elevated excretion of albumin in urine and diminished glomerular filtration rate resulting from abnormalities in the glomerulus. Prolonged diabetes frequently results in diabetic neuropathy, a condition arising from chronic nerve damage [7,8,15]. Diabetic retinopathy is another common complication, impacting around one-third of individuals with high blood pressure and hyperglycemia. Key characteristics include heightened vascular permeability, thickening of the retina, and the growth of new blood vessels, all of which contribute to vision deterioration. Diabetic retinopathy ranks as one of the primary contributors to long-term blindness and visual impairment in people with diabetes [16]. 

In what way does diabetes mellitus (DM) lead to cellular damage and subsequently result in complications in both the microvascular and macrovascular systems?

As mentioned previously, a key characteristic of diabetes mellitus (DM) is the uncontrolled elevation of blood sugar levels, which is closely linked to cellular damage. High glucose levels lead to a rise in the generation of reactive oxygen species (ROS), causing oxidative pressure within the human body [12]. Under typical metabolic conditions, blood sugar undergoes glycolysis and then enters the citric acid cycle in mitochondria. This process produces electron donors like reduced nicotinamide adenine dinucleotide (NADH) and reduced flavin adenine dinucleotide (FADH2), crucial for transferring electrons to molecular oxygen via the electron transport chain (ETC), ultimately leading to the conversion of oxygen into water [11,17]. On the contrary, in unregulated hyperglycemia, the heightened pace of the blood sugar breakdown in the citric acid cycle escalates the entry of substances that donate electrons into the electron transport chain (ETC). This disturbs the ETC, resulting in the formation of superoxide as opposed to water when the voltage gradient rises until it reaches a critical threshold. Consequently, the heightened presence of ROS, like superoxide, in the microvasculature of individuals with diabetes can damage endothelial cells, contributing to various complications affecting both small and large blood vessels.

Metformin and its role in managing diabetes mellitus

Metformin, a derivative of biguanide, is an extensively used oral medication for managing diabetes. Its main use is to treat type 2 diabetes (T2D), particularly in individuals who have excess weight. Unlike insulin, glibenclamide, and chlorpropamide, metformin has shown a notable 30% decrease in mortality and complications related to diabetes [18-20]. Metformin operates by lowering serum glucose levels through various pathways without triggering an increase in insulin secretion. Acting as a substance that increases sensitivity to insulin, it improves cells' sensitivity to insulin [21]. Furthermore, it inhibits the liver's production of glucose by reducing gluconeogenesis rates and having less effect on the breakdown of glycogen. Moreover, metformin enhances the signaling process of insulin and improves blood sugar transportation in muscles. At the same time, it suppresses crucial enzymes related to the production of glucose and the formation of glycogen within the hepatic tissue, particularly when activated by adenosine monophosphate (AMP)-activated protein kinase (AMPK) [18,20,21].

Recent research has underscored metformin's capacity to mitigate both complications affecting small and large blood vessels by impeding cellular damage in both major and minor blood vessels. This protective mechanism is primarily attributed to its influence on the activation of AMP-activated protein kinase in bodily structures and its capability to lower within the cell reactive oxygen species (ROS) [22,23]. Furthermore, metformin has demonstrated potential in reducing the frequency of kidney disease in diabetic individuals by managing reducing oxidative stress and restoring biochemical alterations in kidney tubules, thereby averting tubular damage [24]. Given these compelling findings, metformin has emerged as the most commonly prescribed oral anti-diabetic medication. Its widespread use is attributed to its proven effectiveness, comparatively low-risk profile, and compatibility with other anti-diabetic medications. Currently, an estimated 150 million diabetic individuals globally receive regular metformin treatment [21].

Adverse Effects of Metformin

Metformin generally has minimal side effects, but it does carry the risk of causing lactic acidosis, a serious condition characterized by symptoms such as dizziness, extreme drowsiness, muscle pain, fatigue, chills, pale or bluish skin, rapid or difficult breathing, irregular heartbeat, abdominal discomfort accompanied by diarrhea, nausea, or vomiting. The chances of lactic acidosis increase when metformin is used alongside other conditions that lower oxygen levels in the bloodstream, like a stroke that occurred recently, heart failure due to congestion, or myocardial infarction, as well as excessive alcohol consumption and excessive loss of body fluids [25]. Although excessive lactic acid buildup is not common, gastrointestinal discomfort is a frequently reported side effect in individuals with type 2 diabetes mellitus (T2DM) who are treated with metformin [18]. Another reported negative outcome linked to the diminished absorption of vitamin B12 associated with metformin usage in individuals with type 2 diabetes mellitus (T2DM). The evidence supporting the relationship between metformin usage and lowered vitamin B12 levels varies in its strength [26-28]. Nevertheless, certain aspects of this association require clarification. This review primarily aims to investigate the potential link in the association of metformin usage with vitamin B12 insufficiency.

Metformin and the Occurrence of Vitamin B12 Deficiency

Over the past twenty years, there has been a growing focus on investigating the correlation involving the utilization of metformin and a deficiency in vitamin B12. The initial documentation of vitamin B12 absorption linked to metformin dates back to 1971, as reported by Tomkin et al [29]. Subsequently, numerous experimental studies, observational research, and systematic reviews have shed light on the connection between metformin intake and vitamin B12 deficiency in patients with type 2 diabetes mellitus (T2DM) [26-28,30]. This impact of metformin on the absorption of vitamin B12 has also been noted in individuals with polycystic ovary syndrome (PCOS) who receive metformin treatment. A meta-analysis involving six randomized controlled trials indicated that the use of metformin led to dose-dependent reductions in vitamin B12 levels in patients with T2DM or PCOS [29]. Grasping the exact nature of the association between metformin and vitamin B12 deficiency is vital because of the substantial clinical implications of deficiency in vitamin B12 and its effects on the well-being of people with diabetes. To delve deeper into this connection, it is imperative to possess a comprehensive comprehension of the characteristics of vitamin B12, its absorption, and the process by which metformin could potentially hinder its absorption.

Vitamin B12

Cobalamin, commonly known as vitamin B12, is a hydrophilic vitamin that includes cobalt and acts as an essential co-factor for various metabolic enzymes [31]. All active forms of cobalamin in individuals, like cyanocobalamin, hydroxocobalamin, methylcobalamin, and cobalamin bound to 5-deoxyadenosine, are collectively known as vitamin B12 (adenosyl-Cbl). Furthermore, commercial products offer different formulations of the first three types. Physiologically active forms, adenosyl-Cbl, and methylcobalamin are internally generated from all types of vitamin B12 [30,32,33]. Vitamin B12 plays a crucial role in intracellular enzyme activities linked to the synthesis of DNA, amino acid metabolism, metabolism of fatty acid, red blood cell production, and the effective operation of the central nervous system [31,33]. The uptake of vitamin B12 involves a complex process that relies on various proteins and receptors. Comprehension of this intricate procedure is essential for comprehending the relationship between vitamin B12 malabsorption and additional medications such as metformin. Vitamin B12 obtained from diet is typically bound to proteins. Gastric acid and pepsin in the stomach break down protein-bound vitamin B12. Subsequently, the unbound vitamin attaches to R-binder, which is a glycoprotein found in saliva and gastric secretions, which shields it from the stomach's acidic environment. Pancreatic enzymes degrade the R-binder in the first part of the small intestine, which releases vitamin B12. Gastric intrinsic factor (GIF), which is a protein containing attached sugar molecules, is released by the parietal cells of the stomach and subsequently attaches to the released vitamin B12 to form the complex of intrinsic factor with vitamin B12 [34,35].

This complex undergoes endocytosis through receptors at the end of the small intestine, evading protein degradation and serving as a transporter for vitamin B12. It attaches to the receptor cubilin located in the ileum, which is a protein with attached sugar molecules located on the top surface of cells in the ileum. Certain regions of cubilin interact with the IF-vitamin B12 complex, with the process necessitating calcium cations to enhance. The strength of the complex's attraction to the receptor [36]. Afterward, cells found in the ileum internalize the complex formed by intrinsic factors, vitamin B12, and the cubilin receptor through endocytosis. During endocytosis, the IF separates from cubilin. Inside the lysosome, the IF undergoes breakdown, enabling vitamin B12 to traverse the cell membrane into the interior of the cell [36]. Subsequently, the vitamin moves through the circulation, bound to either transcobalamin-I (TC-I) or TC-II. Approximately 20% to 30% of vitamin B12 present in the bloodstream is attached to the transcobalamin-II protein. Recently assimilated vitamins are captured and conveyed to particular structures through receptor-mediated internalization [34,36].

What mechanisms lead to metformin-induced vitamin B12 malabsorption?

**Figure 2:**
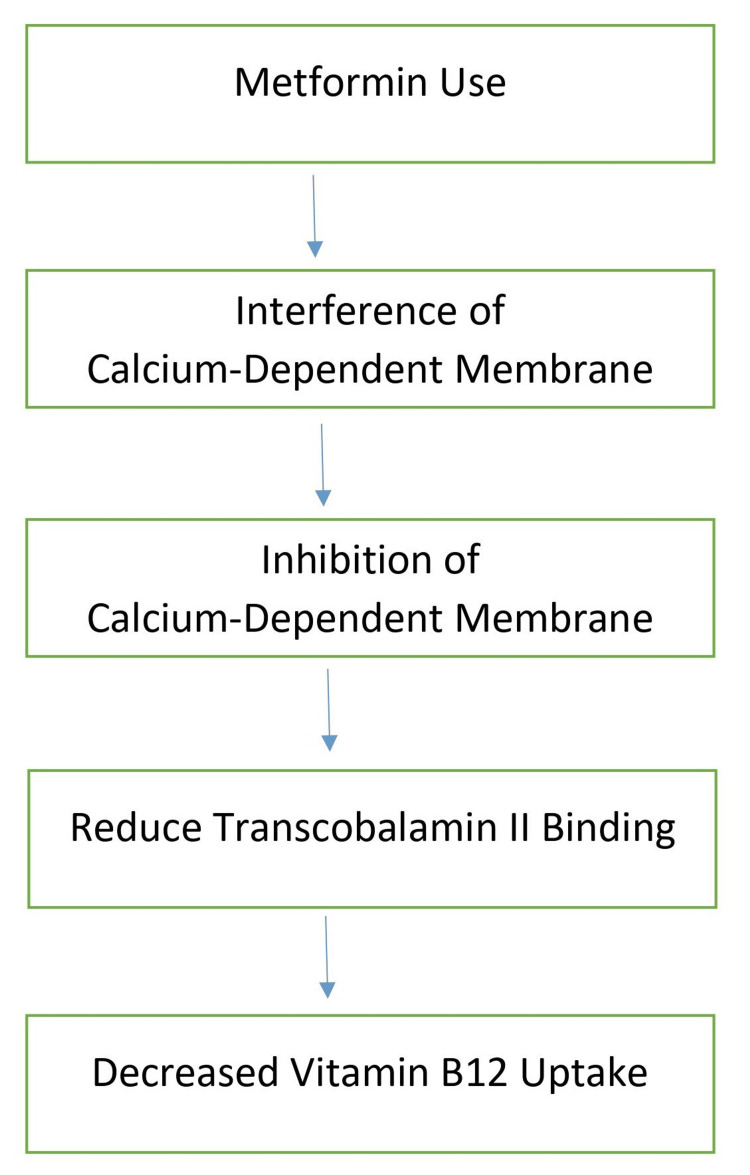
Sequential steps involved in how metformin use can lead to vitamin B12 malabsorption. Note: This image is the author's own creation.

The precise mechanism through which metformin reduces the absorption of vitamin B12 remains somewhat unclear [37]. Several hypotheses have been put forth to elucidate how metformin interferes with vitamin B12 absorption. These theories encompass compromised enterohepatic circulation of B12, elevated storage of vitamin B12 in the liver, diminished production of intrinsic factor (IF), and reduced movement in the intestines coupled with excess growth of bacteria [5,38]. Among these, the most widely acknowledged hypothesis suggests that metformin acts as an antagonist to calcium cations, impeding the calcium-dependent binding of the complex of intrinsic factors and vitamin B12 binding to the cubilin receptor in the ileum. This disruption, as a result, interferes with the cellular uptake mechanism of vitamin B12 [34,38]. One hypothesis proposes that metformin might potentially impart an electrically favorable state at the outer layer of the cubilin receptor membrane [37]. This positive charge might generate repulsive forces on the divalent calcium cations, thereby disturbing the calcium-dependent binding of the IF-vitamin B12 complex to the ileal cubilin receptor, leading to diminished uptake of vitamin B12 [38]. The sequential steps involved in how metformin use can lead to vitamin B12 malabsorption are diagrammatically explained in Figure 2.

Outcomes of vitamin B12 insufficiency in individuals with type 2 diabetes receiving metformin treatment

As previously discussed, numerous observational and experimental studies have indicated an association between prolonged metformin usage and reduced levels of vitamin B12. A lack of vitamin B12 can result in diverse clinical symptoms that greatly affect one's quality of life with type 2 diabetes mellitus (T2DM). This review aims to summarize the main complications related to deficiency in vitamin B12 in T2DM patients with metformin treatment.

Neuropathy

Neuropathy is a significant issue in type 2 diabetes mellitus (T2DM) and is closely associated with vitamin B12 deficiency. Peripheral neuropathy, characterized by weakness, numbness, and pain resulting from nerve damage beyond the brain and spinal cord, is common. Latest research has indicated a potential link between prolonged metformin use and increased incidence of peripheral neuropathy in individuals with T2DM [26,27,39,40]. Intriguingly, one study observed a direct correlation between the duration of the association between metformin treatment and the extent of peripheral neuropathy [26]. The issues extend beyond peripheral neuropathy to include autonomic cardiac neuropathy as well. In a randomized, placebo-controlled trial conducted by Hansen et al. involving 469 individuals with diabetes who had been utilizing insulin for typically 10 years, cardiovascular autonomic neuropathy was assessed through three tests assessing cardiovascular reflexes. Participants were chosen arbitrarily to receive either metformin or a dummy pill. The findings revealed that while vitamin B12 levels remained stable with the placebo over 18 months, they declined with metformin therapy. Furthermore, a metformin group experienced a notable decrease in orthostatic blood pressure, suggesting a deterioration in cardiovascular autonomic neuropathy [41]. Recent studies have connected cardiac autonomic neuropathy with cardiac events, arrhythmias, and sudden death. In particular, one study reported a 3.16-fold rise in cardiovascular disorders (95% confidence interval: 2.42-4.13, P = 0.0001) and a 3.17-fold increase in mortality (95% confidence interval: 2.11-4.78, P = 0.0001) linked to cardiac autonomic neuropathy [42].

Neuropsychiatric Disorders

The reduction in vitamin B12 absorption caused by metformin might impact cognitive function among patients undergoing treatment. Numerous research works have connected decreases in cognitive abilities and certain depressive signs to insufficient levels of vitamin B12 [31]. Porter et al. conducted a cohort study revealing a correlation between metformin usage, decreased levels of vitamin B12 and vitamin B6, and a heightened likelihood of cognitive impairment [43]. Moreover, two recent investigations discovered that individuals receiving metformin treatment and encountering a deficiency of vitamin B12 demonstrated reduced cognitive performance and an increased probability of encountering depressive symptoms [44,45].

Anemia

Since metformin has the potential to induce vitamin B12 deficiency, it may also contribute to the onset of anemia. Vitamin B12 deficiency can lead to the delayed development of erythrocytes and various alterations in their morphology, ultimately causing giant cell anemia. This type of anemia is defined by an irregularity in the maturation of the cell nucleus compared to the cytoplasm, along with abnormal nuclear development in red blood cells. This condition arises due to a deficiency in vitamin B12, combined with insufficient folate levels, which affects DNA synthesis, thereby slowing down the process of cell nucleus duplication and every phase of cell growth [46,47]. Although many studies provide evidence for the observed link between the usage of metformin and deficiency of vitamin B12, there remains uncertainty regarding whether metformin directly causes anemia and, if so, whether this is primarily driven by deficiency of vitamin B12 in individuals with type 2 diabetes mellitus (T2DM) undergoing metformin therapy [48,49]. In this regard, Donnelly and colleagues performed several statistical evaluations utilizing information gathered from a pair of randomized trials conducted in a clinical setting and a study based on observation. Their discovery suggested that metformin use might result in reduced hemoglobin levels, potentially correlating with an elevated likelihood of developing anemia in people with type 2 diabetes mellitus (T2DM) [50]. However, previous well-conducted clinical trials focusing on the correlation between reduced analysis of vitamin B12 levels and metformin usage did not specifically examine the impact of metformin usage on blood-related parameters. Nevertheless, several case reports have associated megaloblastic anemia with extended metformin usage in patients with type 2 diabetes mellitus (T2DM) [5,51].

Management of vitamin B12 deficiency induced by metformin treatment

As previously noted, multiple studies, including clinical trials, observational studies, and meta-analyses, have suggested that long-term use of metformin might lead to vitamin B12 deficiency, which could result in various related complications. To mitigate these risks, it may be necessary to supplement with vitamin B12 [52]. A recent systematic review that included seven clinical trials underscored the importance of supplementing with vitamin B12 in managing or preventing deficiency and neuropathy in individuals with type 2 diabetes mellitus (T2DM) treated with metformin, recommending its inclusion in T2DM management strategies [28]. Likewise, in a recent trial in which participants were randomly assigned, neither they nor the researchers knew who was receiving the treatment and who was receiving the placebo found that administering 1 mg of oral methylcobalamin to individuals with nerve damage due to diabetes treated with metformin for twelve months resulted in enhanced plasma levels of B12 and alleviation of neurophysiological symptoms [53]. Given the usual storage of vitamin B12 in hepatic tissues at 2500 pg., it is generally hepatic reserves of vitamin B12 depletion of these reserves would require the usage of metformin for a minimum of five years. However, additional factors may hasten this depletion, especially in the elderly population, where there is a significant occurrence of atrophic gastritis and the use of acid pump inhibitors. Recent research emphasized the significance of monitoring B12 levels, especially in individuals undergoing prolonged metformin treatment (exceeding four years), particularly when used alongside proton pump inhibitors [5].

## Conclusions

Metformin's potential to induce vitamin B12 deficiency stems from its ability to diminish the absorption of the intrinsic factor (IF) complex via the enteral cubilin receptor in the terminal ileum. This deficiency can lead to various complications, including nerve damage in the extremities, dysfunction of the autonomic nervous system related to the heart, symptoms affecting the brain and nerves, or blood-related disorders. A specific concern involves the emergence or exacerbation of cardiac autonomic neuropathy, which is associated with a higher risk of cardiac irregularities, heart-related incidents, and mortality. Consequently, individuals using metformin are advised to receive yearly evaluations for vitamin B12 insufficiency. If a deficiency is identified, immediate replacement therapy with intramuscular vitamin B12 is advised to replenish the body's stores of the vitamin.
